# Dengue-associated hemophagocytic lymphohistiocytosis in an adult: A case report and literature review: Erratum

**DOI:** 10.1097/MD.0000000000006530

**Published:** 2017-03-24

In the article “Dengue-associated hemophagocytic lymphohistiocytosis in an adult: A case report and literature review”,^[1]^ which appeared in Volume 96, Issue 8 of *Medicine*, the term hemophagocytic lymphohistiocytosis was incorrectly represented as one word throughout the article.
